# Editorial: Exercise, diet, cytokines and obesity

**DOI:** 10.3389/fendo.2024.1527893

**Published:** 2025-01-07

**Authors:** Junhao Huang, Xu Yan, Bing Shen, Liwei Xie, Chia-Hua Kuo

**Affiliations:** ^1^ Guangdong Provincial Key Laboratory of Physical Activity and Health Promotion, Guangzhou Sport University, Guangzhou, Guangdong, China; ^2^ Dr. Neher’s Biophysics Laboratory for Innovative Drug Discovery, State Key Laboratory of Quality Research in Chinese Medicine, Macau University of Science and Technology, Macao, Macao SAR, China; ^3^ Institute for Health and Sport, Victoria University, Melbourne, VIC, Australia; ^4^ Guangdong Institute of Microbiology, Guangdong Academy of Science, Guangzhou, China; ^5^ Laboratory of Exercise Biochemistry, University of Taipei, Taipei, Taiwan

**Keywords:** exercise, diet, cytokines, obesity, overweight

The prevalence of obesity has rapidly increased worldwide, which represents a major global health challenge with important clinical implications (1). As a complex disease characterized by abnormal or excessive fat accumulation, obesity increases the risk of many other diseases and health problems, such as metabolic diseases, cardiovascular diseases, cognitive dysfunction, sleep apnea, cancers, etc. In recent years, efforts have been made in investigating the lifestyle strategies which may prevent or treat obesity. Exercise have been shown to reduce the risk of obesity. However, it is still unclear which specific modality of exercise may have a more significant effect on reducing obesity. More studies are still needed to investigate the optimal exercise mode, intensity, and frequency, as well as explore the underlying mechanisms behind the changes in obesity. Besides exercise, diet is another important factor that may affect obesity. Furthermore, growing evidence has shown that exercise can increase the release of cytokines, such as IL-6, FGF21, and myostatin, from skeletal muscle or other organs into the blood. These cytokines can modulate adipose tissue metabolism (2). However, there is still much to explore to fully understand the roles of exercise-induced cytokines and the underlying mechanisms involved, especially in relation to the treatment and prevention of obesity.

In this Research Topic, a total of 7 papers were published to cover the above-mentioned aspects among different populations.

One cross-sectional study (Zhu et al.) examined whether cardiorespiratory fitness mediates the association between body fat rate and executive function in young adults and concluded that cardiorespiratory fitness of young adults plays a partial mediating role between body fat rate and executive function. Strategies to motivate overweight youth to exercise need to be tested in order to enhance their cardiorespiratory fitness and mitigate the detrimental effects of obesity and overweight on executive function. One prospective study (Cai et al.) collected the physical examination results in 2019 to 2022 to investigate the impact of lockdowns on weight%, BMI%, overweight rate, obesity rate, and combined overweight and obesity rate of children in China aged 3-6 years during COVID-19 pandemic period, and the results showed that the lockdowns in COVID-19 pandemic promoted obesity of kindergarten children. These two studies highlight the importance of physical exercise in reducing the risk of obesity. Another cross-sectional study (Li et al.) collected data from NHANES 2017–2020 to investigate the joint associations between lifestyle exposomes, including sleep duration, metabolic equivalent of task, Healthy Eating Index-2015 score, alcohol consumption, and smoke exposure, and metabolic dysfunction-associated fatty liver disease. This study demonstrated significant associations between metabolic dysfunction-associated fatty liver disease and single and joint exposures to sleep duration, metabolic equivalent of task, and Healthy Eating Index-2015, and identified physical activity as the most important lifestyle factor.

Besides observational studies, one intervention study (Wang et al.) compared the acute effects of three exercise modalities including tabata, high-intensity interval training (HIIT), and moderate-intensity continuous training (MICT) on the energy expenditure and substrate metabolism in male university students with overweight/obesity. This study reported that, during both the exercise and recovery phases, tabata exhibited a significantly higher fat oxidation rate than HIIT and MICT and suggested that tabata can be used as an efficient short-term weight loss exercise program for male college students with overweight/obesity.

Besides the original studies, two meta-analyses were also published in this Research Topic. One meta-analysis (Wang et al.) compared the effects of various training interventions including aerobic exercise, resistance training, combined aerobic and resistance training, and HIIT on metabolic indicators in adults with overweight/obesity. This study included a total of 28 randomized controlled trials (RCTs) and concluded that aerobic exercise is the optimal exercise type for reducing body weight and BMI, while HIIT exerts the most beneficial effects on improving body composition, cardiorespiratory fitness, and metabolic abnormalities in adults with overweight/obesity. Another meta-analysis (Gao et al.) included 10 RCTs to investigate the effects of Baduanjin on weight reduction in individuals with overweight/obesity. The study reported that Baduanjin is effective in managing obesity and facilitating weight loss in individuals with overweight/obesity. However, further well-designed RCTs are still necessary to provide more robust evidence in the future.

The last paper in this Research Topic (Wen et al.) focused on the relationship between an adipokine called Apelin-13 and metabolic diseases and exercise. The review summarized the recent studies on the relationship between Apelin-13 and related metabolic diseases as well as the regulatory effects of exercise on Apelin-13 and concluded that more studies are still needed to investigate the effects of exercise with different types, intensities, and amounts on Apelin-13 in the future.

In summary, there are large varieties among the included studies in this Research Topic. However, most of the studies further support the benefits of physical activity and on reducing the risk of obesity among different populations. Additionally, diet is another important factor that may affect obesity. However, the effects of combined exercise and diet on obesity are still unclear so far. Unfortunately, no related studies were included in this Research Topic. In addition, there is only one study related to cytokines in this Research Topic. Obviously, further research is needed to explore the relationship between lifestyle interventions and obesity as well as the underlying mechanisms.
